# Effects of Dietary Active Dry Yeast Supplementation on Lactation Performance and Rumen Fermentation in Holstein Cows

**DOI:** 10.3390/ani16193049

**Published:** 2026-09-28

**Authors:** Jean Bosco Nzeyimana, Yapeng Hu, Maocheng Jiang, Dian Wang, Sijia Liu, Zhao Zhuo, Caiyun Fan, Jianbo Cheng

**Affiliations:** 1 College of Animal Science and Technology, Anhui Agricultural University, Hefei 230036, China; nzeyimana26@gmail.com (J.B.N.); 18567032653@163.com (Y.H.);; 2 National Center of Technology Innovation for Dairy, Hohhot 010010, China; 3 Animal Nutrition and Feed Research Center, Anhui Agricultural University, Hefei 230036, China

**Keywords:** dairy cows, active dry yeast, milk fatty acid profile, apparent digestibility

## Abstract

Active dry yeast (ADY) as a feed additive has been proven to have functions such as enhancing feed digestion and utilization rates, strengthening the body’s immunity, alleviating stress and improving rumen fermentation. This experiment mainly explores the effects of a new type of ADY on the production performance, rumen fermentation and nutrient digestibility of dairy cows. A total of 100 Holstein cows with similar milk yield (46.9 ± 8.5 kg/d), days in milk (66.7 ± 5.4 d), and parity (2.5 ± 1.0) were selected and randomly allocated into four groups (25 cows per group). The treatments were as follows: (1) Control group (CON), cows fed the basal diet only; (2) ADY I group (ADY I), basal diet supplemented with 5 g/d of common ADY (RF7, 1 × 10^10^ CFU/g) per cow; (3) Low ADY II group (LADY II), basal diet supplemented with 5 g/d of Actisaf Sc48 (viable yeast cell concentration is 1 × 10^10^ CFU/g) per cow; (4) High ADY II group (HADY II), basal diet supplemented with 10 g/d of Actisaf Sc48 per cow. The 10-week experimental period consisted of a 1-week adaptation period followed by a 9-week formal experimental period. The results showed that, compared with the CON group, mean dry matter intake (DMI) increased by 3.48%, 5.35%, and 6.96% in the ADY I, LADY II, and HADY II groups, respectively (*p* < 0.05), with a significant treatment × time interaction (*p* < 0.001). Compared with the CON and ADY I groups, the HADY II group showed increases in milk yield of 5.76% and 4.76% and in apparent crude protein (CP) digestibility of 5.22% and 3.17%, respectively (*p* < 0.05). Milk lactose concentration was 3.43% higher in the HADY II group than in the ADY I group (*p* < 0.05). Additionally, milk linolelaidic acid concentration was lower in LADY II and HADY II than in CON (*p* < 0.05). Compared with the CON group, the LADY II and HADY II groups showed increases in ruminal total volatile fatty acid (TVFA) concentrations of 42.11% and 44.72% and in acetate concentrations of 45.76% and 46.71%, respectively (*p* < 0.05). In this trial, the group receiving ADY II at 10 g/cow/day showed the best overall performance. However, this finding does not establish 10 g/cow/day as the optimal inclusion level for this product. Future studies should compare the two products at matched doses and integrate multi-omics analyses with functional validation and economic evaluation to clarify their mechanisms of action and guide practical supplementation.

## Simple Summary

This study compared two active dry yeast products in 100 lactating Holstein cows over ten weeks. Cows received a basal diet without yeast, conventional active dry yeast (RF7) at 5 g/cow/day, or Actisaf Sc48 at 5 or 10 g/cow/day. Compared with the control and conventional yeast groups, cows receiving Actisaf Sc48 at 10 g/cow/day had higher milk yield and apparent crude protein digestibility. Milk lactose content was higher with Actisaf Sc48 at 10 g/cow/day than with conventional yeast, but did not differ significantly from the control. Both Actisaf Sc48 groups had higher concentrations of total volatile fatty acids and acetate in the rumen than the control and conventional yeast groups. Feed intake differed among treatments, and these differences varied over time.

## 1. Introduction

Active dry yeast (ADY) is a well-established dietary additive in ruminant nutrition, with a robust body of empirical evidence supporting its multifaceted biological benefits [1,2]. A study showed that supplementing the diets of 50 Holstein cows with 10 g of Actisaf Sc47 per cow per day (1 × 10^11^ CFU/day) increased milk yield from 47.5 to 50.1 kg/day, while increasing apparent dry matter digestibility from 77% to 80% and apparent neutral detergent fiber (NDF) digestibility from 62% to 66% [3]. Another study evaluated supplementation with 10 g/day of ADY (*Saccharomyces cerevisiae*, CNCM I-4407) in 117 multiparous Holstein cows. The results showed that milk yield was higher in the 10 g/d group than in the control group (52.0 vs. 48.5 kg/day) [4]. Furthermore, in a study involving 33 heat-stressed Holstein cows, supplementation with 20 g of *S. cerevisiae* Y03-0 per cow per day increased ruminal total volatile fatty acid (TVFA) concentrations from 82.73 to 92.33 mmol/L and acetate concentrations from 53.45 to 60.22 mmol/L [5]. These studies provide a basis for evaluating the effects of specific yeast products at specific inclusion levels based on milk yield, apparent nutrient digestibility, and ruminal fermentation.

Nevertheless, a growing body of research highlights that the functional efficacy of yeast is not uniform. A study showed that supplementing Holstein cow diets with 5 g of BIOSAF Sc47 per cow per day significantly increased ruminal TVFA concentration from 85.3 to 99.4 mmol/L [6]. In contrast, another study found that supplementation with Vistacell at 0.125 g/kg of dietary dry matter (DM) did not significantly affect ruminal TVFA concentration in Holstein cows [7]. Furthermore, supplementation with *Saccharomyces cerevisiae* YE1496 at 5.7 × 10^7^ CFU/day significantly increased milk yield in lactating dairy cows compared with the control (31.7 vs. 29.6 kg/day), whereas supplementation at 6.0 × 10^8^ CFU/day did not significantly affect milk yield (30.0 vs. 29.6 kg/day) [8]. These findings indicate that the effects of yeast supplementation on dairy cows vary among yeast additives and among supplementation levels of the same yeast. Therefore, this study aims to evaluate and compare the effects of a novel ADY feed additive (Actisaf Sc48) with those of conventional ADY (RF7) on milk composition, apparent nutrient digestibility, and rumen fermentation characteristics in lactating dairy cows, and to investigate whether the ADY exhibits dose-dependent effects through different additive levels, thereby providing reference for the application of yeast additives in ruminant animals.

## 2. Materials and Methods

### 2.1. Animal Ethics

This experiment was approved by the Laboratory Animal Management and Animal Ethics Committee of Anhui Agricultural University (No. SYDW-P20220308061).

### 2.2. Experimental Materials

Two active dry yeast products were used in this experiment. Active dry yeast strain I (ADY I), designated RF7, was a strain of *Saccharomyces cerevisiae* manufactured by a company in China. It was supplied as a light yellowish-brown powder with a labeled viable cell concentration of 1.0 × 10^10^ CFU/g. The product batch number was 2021120301, and its shelf life was two years. Active dry yeast II (ADY II), Actisaf Sc48, was manufactured by Phileo, a company of the Lesaffre Group (Marcq-en-Baroeul, France). It contained a proprietary *S. cerevisiae* strain with a labeled viable cell concentration of 1.0 × 10^10^ CFU/g. The product was supplied in a highly concentrated microgranular form and manufactured using Boost Technology™, intended for incorporation into non-pelleted premixes and concentrates used in total mixed ration (TMR) diets. According to the manufacturer, this technology enhances product stability in the presence of organic acids and other acidifiers. The product batch number was 22B0105, and its shelf life was two years. Both products were stored in a cool, dry place protected from light, heat, and moisture until use. Before the experiment, viable cell concentrations were verified by plate counting, and the measured concentrations of both products were consistent with their labeled values.

### 2.3. Animals and Experimental Design

The experiment was conducted from March to June 2022 at the Liuchen Dairy Farm of Youran Animal Husbandry, Hefei, China. During the experimental period, the ambient temperature in the barn ranged from 12.5 to 28.3 °C, the relative humidity ranged from 55% to 78%, and the average daily temperature–humidity index (THI) remained below 68 throughout the study period.

A total of 100 healthy lactating Holstein cows were selected, with an initial average daily milk yield of 46.9 ± 8.5 kg, 66.7 ± 5.4 days in milk, and a parity of 2.5 ± 1.0. Cows with mastitis, lameness, gastrointestinal disorders, infectious diseases, abnormal physiological conditions, or those that had received veterinary drug treatment within 30 d before the experiment were excluded. Cows were blocked according to initial milk yield, days in milk, and parity and were then randomly assigned to four treatment groups (*n* = 25 per group): (1) control (CON), fed a basal TMR; (2) ADYI, fed the basal TMR supplemented with RF7 at 5 g/cow/day; (3) low-dose ADYII (LADYII), fed the basal TMR supplemented with Actisaf Sc48 at 5 g/cow/day; and (4) high-dose ADYII (HADYII), fed the basal TMR supplemented with Actisaf Sc48 at 10 g/cow/day. No significant differences in the major production or physiological characteristics were observed among groups before treatment allocation.

The experiment lasted for 10 weeks, including a 1-week adaptation period followed by a 9-week experimental period. A 7-day adaptation period allows animals to acclimate to the trial pen and achieve stable feed intake, reducing stress. Cows were housed in tie-stall barns, with each cow individually tethered in a separate stall. Feed offered and refusals were weighed and recorded for each cow on three consecutive days each week. Daily dry matter intake (DMI) was calculated as the amount of feed offered multiplied by its dry matter proportion minus the number of refusals multiplied by their dry matter proportion. Cows had free access to water and feed. TMR was offered three times daily at 08:30, 15:30, and 24:00, and the amount offered was adjusted according to feed intake to maintain approximately 5% refusals. Cows were milked four times daily at 09:00, 15:00, 19:00, and 24:00. The health status of all cows was monitored daily throughout the experiment. All 100 cows completed the entire experimental period without removal or dropout. Production performance data, including milk yield, were analyzed using all cows in each treatment (*n =* 25 per treatment). For blood, rumen fluid, and other biological sample collections, 15 cows were randomly selected from each treatment (*n =* 15 per treatment).

The TMR was formulated according to the National Research Council (NRC) recommendations for dairy cattle [9]. The ingredient composition and nutrient levels of the diet, expressed on a DM basis, are presented in Table 1. During the experiment, the four groups were fed identical TMR diets, with yeast administered only as an additive and not replacing any feed ingredients. Feed nutrient composition was determined through laboratory analysis. Feed, milk, rumen fluid, and other samples were collected at regular intervals according to the experimental design and stored and analyzed following established procedures.

### 2.4. Sample Collection and Preparation

#### 2.4.1. Collection and Preparation of Feed Samples

Feed samples were collected on three consecutive days each week during the trial. For each treatment, a five-point sampling method was used to collect primary subsamples from multiple locations—front, middle, rear, and both sides of the feed trough. The primary subsamples were thoroughly mixed, then reduced using the quartering method, resulting in approximately 500 g of a representative composite sample for analysis. The composite samples were oven-dried at 65 °C for 48 h to determine DM content. The dried samples were subsequently ground through a 1-mm screen using a Wiley mill (Model 4; Arthur H. Thomas, Philadelphia, PA, USA) for chemical analysis. Feed refusals were collected simultaneously, mixed thoroughly, subsampled, dried, and weighed to calculate dry matter intake (DMI). Individual daily DMI was calculated as the difference between DM offered and DM refused.

#### 2.4.2. Collection and Preparation of Milk Samples

The milk yield of each cow was recorded daily throughout the experimental period. On the first day of each week, milk samples were collected from each cow at each of the four daily milkings. Immediately after milk sample collection, potassium dichromate (analytical grade, catalog number 10016619, Hushi, Sinopharm Chemical Reagent Co., Ltd., Shanghai, China) was added as a preservative. Daily composite milk samples were prepared by pooling subsamples in a volume ratio (3:2:2:3) corresponding to the milk yield proportion of each milking to obtain a representative daily milk sample, following the principle of proportional composite milk sampling [10]. Each composite milk sample was divided into two equal portions: one was stored at 4 °C for milk composition analysis, and lipid extraction and fatty acid analysis were performed within 72 h after sampling, and the other at −20 °C for milk fatty acid profile analysis.

#### 2.4.3. Collection and Preparation of Ruminal Fluid Samples

Two hours after morning feeding on the last day of the trial period, rumen fluid was collected from 15 randomly selected cows in each group. Rumen fluid was collected using a rumen fluid sampler (Kelibo, Co., Ltd., Wuhan, China), and a sampling catheter was inserted into the rumen through the mouth and esophagus; 200 mL of rumen fluid collected at the beginning of the collection was discarded, and approximately 200 mL of rumen fluid was subsequently collected. The pH of the rumen fluid samples was measured immediately after collection using a portable pH meter (PHB-4; Yidian Scientific Instrument Co., Ltd., Shanghai, China). Each rumen fluid sample was filtered through four layers of gauze, and the filtrate was stored at −20 °C for analysis of ammonia nitrogen (NH_3_-N), microbial protein (MCP), and volatile fatty acids (VFAs).

#### 2.4.4. Collection and Preparation of Fecal Samples

During the final week of the experiment, fecal samples were collected from 15 cows per treatment group on three consecutive days. Fecal samples collected over three consecutive days were pooled within each cow using equal fresh weights. The composite samples were dried to constant weight in a forced-air oven at 65 °C and subsequently ground to pass through a 1-mm screen. The dried samples were analyzed for ash, total nitrogen (N), NDF, acid detergent fiber (ADF), ether extract (EE), and acid-insoluble ash (AIA). AIA was used as an internal marker, and the apparent total-tract digestibility of each nutrient was calculated based on the concentrations of AIA and the corresponding nutrient in the diets and feces [11]. The calculation is expressed as follows:

$$Digestibility(\%)=100\times [1-\frac{{AIA}_{d}}{{AIA}_{f}}\times \frac{N_{f}}{N_{d}}]$$

AIA and N denote marker and nutrient concentrations, respectively, and the subscripts d and f identify diet and feces. All concentrations are expressed on the same DM basis.

#### 2.4.5. Collection and Preparation of Blood Samples

On the final day of the experiment, approximately one hour before morning feeding, 15 cows were randomly selected from each group. Ten milliliters of blood was collected from each cow via the tail vein into vacuum tubes containing EDTA. Immediately after collection, the tubes were gently inverted to ensure thorough mixing of the blood with the anticoagulant. The anticoagulated whole-blood samples were stored and transported to the laboratory at 2–8 °C without centrifugation. Before analysis, the samples were allowed to return to room temperature and thoroughly mixed.

### 2.5. Determination of Indicators and Methods

#### 2.5.1. Milk Composition Analysis

Milk fat, protein, lactose, and total solids contents and milk urea nitrogen (MUN) concentration were determined using a mid-infrared milk analyzer (MilkoScan FT120; Foss Electric, Hillerød, Denmark). Milk somatic cell count (SCC) was determined using a somatic cell counter (Fossomatic 5000; Foss Analytical A/S, Hillerød, Denmark).

#### 2.5.2. Milk Fatty Acid Analysis

Milk samples were prepared with reference to GB 5009.168–2016 (National Food Safety Standard—Determination of Fatty Acids in Foods) [12]. Total lipids were extracted using chloroform–methanol (2:1, *v*/*v*), followed by alkaline hydrolysis and methylation with boron trifluoride–methanol under constant-temperature water-bath heating. The resulting fatty acid methyl esters (FAMEs) were purified and passed through a 0.22-μm solvent-compatible syringe filter (Jinteng, Tianjin, China) before analysis. FAMEs were analyzed using an Agilent 7890B gas chromatograph equipped with a flame ionization detector (FID) and ChemStation software (B.04.03-SP1, Agilent Technologies, USA). Separation was performed on an SP-2560 capillary column (100 m × 0.25 mm internal diameter, 0.25 μm film thickness; Sigma-Aldrich, St. Louis, MO, USA). High-purity nitrogen (≥99.999%) was used as the carrier gas at a constant flow rate of 0.8 mL/min. The oven temperature was maintained at 100 °C for 5 min, increased to 225 °C at 4 °C/min, and then increased to 240 °C at 2 °C/min and held for 17 min. The injection volume was 1.0 μL, with a split ratio of 50:1. The injector and detector temperatures were maintained at 250 and 280 °C, respectively. Individual FAMEs were identified by comparing their retention times with those of the Supelco 37-Component FAME Mix (catalog no. CRM47885), using a relative retention-time tolerance of ±0.5%. Quantification was performed using the same reference mixture as an external standard. Concentrations in the original milk samples were calculated as follows:

$$C_{i}=\frac{A_{s,i}\times C_{std,i}\times V_{f}}{A_{std,i}\times V_{s}}$$

where C_i_ is the analyte concentration in the original milk sample (mg/mL); A_s,i_ and A_std,i_ are the sample and standard peak areas, respectively; C_std_,_i_ is the analyte concentration in the standard solution (mg/mL); V_f_ is the final extract volume (mL); and V_s_ is the initial milk sample volume (mL).

#### 2.5.3. Feed and Fecal Sample Analysis

The moisture content of feed and fecal samples was determined according to GB/T 6435–1986 [13], and DM content was calculated accordingly. CP and EE contents were determined according to GB/T 6432–1994 [14] and GB/T 6433–1994 [15], respectively. Crude ash content was determined gravimetrically following overnight incineration in a muffle furnace at 550 °C, with reference to GB/T 6438–1992 [16]. Organic matter (OM) content was calculated as 100 minus the ash percentage, with both OM and ash expressed on a DM basis. NDF and ADF contents were determined according to the methods described by Van Soest et al. (1991) [17]. The apparent digestibility of each nutrient was calculated using AIA as an internal marker, based on the concentrations of AIA and the corresponding nutrient in the diets and feces.

#### 2.5.4. Ruminal Fermentation Analysis

MCP content was determined using the Coomassie Brilliant Blue G-250 colorimetric method [18]. The staining solution was prepared with Coomassie Brilliant Blue G-250, 95% ethanol and 85% phosphoric acid, made up to volume, filtered, and stored at 4 °C in the dark for a maximum of one week. Microbial pellets were obtained by sample vortexing followed by step-wise low-temperature centrifugation. Protein was extracted from pellets in NaOH solution via boiling-water bath, and the collected supernatant was appropriately diluted after centrifugation. Both diluted samples and serially diluted bovine serum albumin (BSA) standards were mixed with Coomassie Brilliant Blue staining solution, allowed to develop color for 2 min at room temperature, and absorbance was measured at 595 nm with a spectrophotometer (Mapada, Shanghai, China). The MCP concentration of samples was calculated against the established standard curve. NH_3_-N was measured using the phenol–hypochlorite method [19]. Reagents included phenol solution, sodium hypochlorite solution, and ammonium chloride stock standard solution. A 6-point external standard curve (0–10 mg N/L) was prepared by serial dilution of ammonium chloride stock. Distilled water was used as reagent blank, and blank absorbance was subtracted from sample absorbance. Thawed ruminal fluid was centrifuged at 12,000 rpm for 10 min; supernatant was diluted and reacted with phenol and hypochlorite reagents under alkaline conditions for color development. Absorbance was read at 630 nm.

VFAs in ruminal fluid were analyzed using a GC-2014 gas chromatograph (Shimadzu Corporation, Kyoto, Japan) equipped with an FID. After thawing, samples were centrifuged at 12,000 rpm for 10 min. A 1-mL aliquot of the supernatant was thoroughly mixed with 0.2 mL of a solution containing crotonic acid and metaphosphoric acid. The mixture was passed through a syringe filter before injection using an autosampler. Separation was performed on a WondaCap WAX capillary column (30.0 m × 0.25 mm internal diameter; 0.25 μm film thickness; Shimadzu Corporation, Kyoto, Japan). The injector and FID temperatures were maintained at 200 and 220 °C, respectively. The oven temperature was programmed to increase from 80 to 170 °C at 15 °C/min. Crotonic acid served as the internal standard.

#### 2.5.5. Blood Sample Analysis

Hematological parameters were determined using a fully automated hematology analyzer (XT-4000i; Sysmex, Kobe, Japan). The analyzer, including its three-part leukocyte differential, had been validated for bovine blood [20]. The parameters assessed included white blood cell count (WBC), lymphocyte count (Lymph), monocyte count (Mon), granulocyte count (Gran), red blood cell count (RBC), hemoglobin concentration (HGB), hematocrit (HCT), mean corpuscular volume (MCV), mean corpuscular hemoglobin (MCH), mean corpuscular hemoglobin concentration (MCHC), red cell distribution width (RDW), platelet count (PLT), mean platelet volume (MPV), platelet distribution width (PDW), and plateletcrit (PCT). The blood results represent endpoint group comparisons rather than changes from baseline.

### 2.6. Statistical Analysis

Experimental data were entered and organized using Excel 2019 (Microsoft, Redmond, WA, USA) and statistically analyzed using IBM SPSS Statistics 26.0 (IBM, Armonk, NY, USA). The normality of model residuals was assessed using the Shapiro–Wilk test, and homogeneity of variance among treatment groups was evaluated using Levene’s test.

Milk yield, milk composition, DMI and milk fatty acid composition were analyzed using two-way repeated-measures analysis of variance (ANOVA). Individual lactating cows were used as the repeated-measures experimental units, with treatment as the between-group factor and measurement time as the within-group factor; the model included treatment, time, and the treatment × time interaction effect. Sphericity was assessed using Mauchly’s test. When the sphericity assumption was violated, the Greenhouse–Geisser correction was applied to the degrees of freedom for tests of the time and treatment × time effects. Significant interactions were further examined using simple-effects analyses, with Bonferroni adjustment for multiple comparisons. When the interaction was not significant but the main effect of treatment was significant, pairwise comparisons among treatments were performed using Tukey’s honestly significant difference (HSD) test. Blood parameters, apparent nutrient digestibility, and ruminal fermentation parameters were analyzed using one-way ANOVA to assess treatment effects. When the treatment effect was significant, multiple comparisons were performed using Tukey’s HSD test. The dataset used in this study has been verified and contains no missing observations.

All data are presented as means and standard errors of the mean (SEM). Differences were considered statistically significant at *p* < 0.05 and highly significant at *p* < 0.01, whereas 0.05 < *p* < 0.10 was considered to indicate a trend.

## 3. Results

### 3.1. Effects of ADY on Milk Yield and Milk Composition of Dairy Cows

Milk yield was higher in HADYII than in CON and ADYI (*p* < 0.05), with increases of 5.76% and 4.76%, respectively. Milk yield varied over time (*p* < 0.001), but the treatment × time interaction was not significant (*p* = 0.957). Averaged across the experimental period, DMI was higher in ADYI, LADYII, and HADYII than in CON (*p* < 0.05), by 3.48%, 5.35%, and 6.96%, respectively. Mean DMI was also higher in HADYII than in ADYI (*p* < 0.05). However, both the time effect and the treatment × time interaction were significant (*p* < 0.001), indicating that treatment differences in DMI varied over time. Milk lactose concentration was 3.43% higher in HADYII than in ADYI (*p* < 0.05). A time effect was detected for lactose concentration (*p* = 0.002), whereas the treatment × time interaction was not significant (*p* = 0.224). Milk fat, protein, and total solids concentrations, MUN, and SCC did not differ significantly among treatments (*p* > 0.05). Milk protein concentration and MUN varied over time (*p* < 0.001), whereas the time effect on SCC did not reach statistical significance (*p* = 0.074). No significant treatment × time interactions were detected for these variables (*p* > 0.05). (Table 2)

### 3.2. Effects of ADY on Milk Fatty Acids

For saturated fatty acids (SFA), butyric acid and undecanoic acid concentrations were higher in ADYI than in the other groups (*p* < 0.05). Compared with CON, the increases were 144.44% and 52.38%, respectively. Both LADYII and HADYII had lower concentrations of caproic, caprylic, capric, myristic, Pentadecenoic, palmitic, heptadecanoic, and arachidic acids than CON and ADYI (*p* < 0.05). Relative to CON, the reductions in LADYII and HADYII, respectively, were 56.63% and 69.28% for caproic acid, and 48.89% and 49.33% for caprylic acid. Corresponding reductions were 33.63% and 32.20% for capric acid, 34.64% and 31.27% for myristic acid, and 43.93% and 35.51% for Pentadecenoic acid. Palmitic acid concentrations were lower by 36.33% and 33.03%, heptadecanoic acid by 40.00% and 35.00%, and arachidic acid by 30.00% in both groups. Compared with ADYI, lauric acid concentrations were lower by 38.43% in LADYII and 37.21% in HADYII (*p* < 0.05). Compared with CON, stearic, heneicosanoic, and behenic acid concentrations were also lower in both LADYII and HADYII (*p* < 0.05). The respective reductions were 31.68% and 33.04% for stearic acid, 28.57% in both groups for heneicosanoic acid, and 32.35% and 29.41% for behenic acid. Tridecanoic acid concentrations were lower in LADYII than in CON and ADYI, by 43.33% and 56.41%, respectively (*p* < 0.05). HADYII had a 48.72% lower tridecanoic acid concentration than ADYI (*p* < 0.05). Lignoceric acid concentrations were 22.22% lower in LADYII than in both CON and ADYI (*p* < 0.05). Tricosanoic acid concentrations did not differ significantly among treatments (*p* > 0.05) (Table 3).

For monounsaturated fatty acids (MUFA), myristoleic, palmitoleic, cis-10-heptadecenoic, and oleic acid concentrations were lower in LADYII and HADYII than in CON and ADYI (*p* < 0.05). Relative to CON, the reductions in LADYII and HADYII, respectively, were 35.80% and 40.34% for myristoleic acid, and 37.50% and 34.38% for palmitoleic acid. Corresponding reductions were 46.88% and 34.38% for cis-10-heptadecenoic acid, and 37.58% and 38.40% for oleic acid. The concentration of cis-11-eicosenoic acid was 34.62% lower in both LADYII and HADYII than in CON (*p* < 0.05). No significant treatment differences were observed for cis-10-pentadecenoic, elaidic, erucic, or nervonic acid concentrations (*p* > 0.05) (Table 3).

For polyunsaturated fatty acids (PUFAs), linoleic, γ-linolenic, α-linolenic, cis-8,11,14-eicosatrienoic, and eicosapentaenoic acid concentrations were lower in LADYII and HADYII than in CON and ADYI (*p* < 0.05). Relative to CON, the reductions in LADYII and HADYII, respectively, were 31.92% and 29.56% for linoleic acid, and 32.91% and 27.85% for γ-linolenic acid. The concentration of α-linolenic acid was lower by 47.06% in both groups. Corresponding reductions were 35.14% and 27.03% for cis-8,11,14-eicosatrienoic acid, and 33.33% and 26.67% for eicosapentaenoic acid. Linolelaidic acid concentrations were lower in LADYII than in CON and ADYI, by 37.04% and 26.72%, respectively (*p* < 0.05). HADYII had a 34.81% lower linolelaidic acid concentration than CON (*p* < 0.05). The concentration of cis-11,14,17-eicosatrienoic acid was lower in HADYII than in CON and ADYI, by 37.50% and 28.57%, respectively (*p* < 0.05). No significant treatment differences were observed for cis-11,14-eicosadienoic, arachidonic, cis-13,16-docosadienoic, or docosahexaenoic acid concentrations (*p* > 0.05). These comparisons describe means across the experimental period. Significant time effects were detected for most fatty acids, and treatment × time interactions were significant for all except elaidic and nervonic acids (*p* < 0.05) (Table 3).

### 3.3. Effects of ADY on Apparent Total-Tract Digestibility

Compared with the CON and ADY I groups, the HADY II group showed a significant increase in apparent CP digestibility (*p* < 0.05), improving by 5.22% and 3.17% over CON and ADY I, respectively. There were no significant differences among all groups in the apparent digestibility of EE, NDF, and ADF (*p* > 0.05). (Table 4)

### 3.4. Effects of ADY on Rumen Fermentation

Compared with the CON and ADY I groups, the LADY II and HADY II groups showed significantly higher TVFA concentrations (*p* < 0.05), increasing by 42.11% and 44.72%, respectively, compared to CON, and by 30.82% and 33.22%, respectively, compared to ADY I. The acetate concentrations in the LADY II and HADY II groups were also significantly higher than those in the CON and ADY I groups (*p* < 0.05), increasing by 45.76% and 46.71%, respectively, compared to CON, and by 32.71% and 33.58%, respectively, compared to ADY I. No significant differences were observed among the groups in pH, NH_3_-N, MCP, propionate, butyrate, isobutyrate, or acetate-to-propionate ratio (A/P) (*p* > 0.05) (Table 5).

### 3.5. Effects of ADY on Hematological Profile

Compared with the ADY I group, the LADY II and HADY II groups showed a significant reduction in WBC (*p* < 0.05), decreasing by 28.31% and 36.39%, respectively. Meanwhile, Gran levels in the LADY II and HADY II groups were significantly lower than those in the ADY I group (*p* < 0.05), decreasing by 32.78% and 39.47%, respectively. Differences in Mon among treatments tended to be significant (*p* = 0.060), while differences in PDW showed a trend (*p* = 0.090). No significant differences were observed among treatments in Lymph, Lymph%, Mon%, Gran%, RBC, HGB, HCT, MCV, MCH, MCHC, RDW, PLT, MPV, or PCT (*p* > 0.05). (Table 6)

## 4. Discussion

Milk production and milk quality are core indicators of dairy cow production performance and are directly related to the economic benefits of the dairy farm [21]. DMI is a key indicator determining the energy and nutrient intake of dairy cows, directly related to milk production, milk quality (such as milk fat percentage and milk protein percentage), and the health status of dairy cows [22,23]. In this study, the addition of ADY II increased the milk production, DMI and lactose content in the milk of dairy cows. Research has found that supplementing ADY can significantly increase the milk production of lactating dairy cows, while also enhancing the concentrations of milk fat and lactose [24]. This is consistent with the findings of this study. In addition, under heat stress conditions, supplementing active yeast significantly increased the DMI of dairy cows compared to the CON group [25]. However, in a trial lasting from 28 d prepartum to 13 weeks postpartum, supplementation with yeast culture had no significant effect on DMI both before and after calving [26]. This might be caused by differences in yeast activity, supplementary dosage, physiological stage of dairy cows, and feeding environment [27,28,29]. In this study, supplementation with ADY II significantly increased milk yield and DMI. Higher milk yield can reduce production costs per unit of milk, increase raw milk output at a fixed herd size, and reduce nitrogen and methane emissions per unit of milk produced [30]. The simultaneous improvement in feed intake and lactation performance represents a favorable physiological response in dairy cows. The concurrent increases suggest that greater feed intake may have helped support the nutrient demands of higher milk production.

Milk fat contains a complex mixture of fatty acids in natural foods [31]. So far, over 400 types of fatty acids have been identified, among which about 12 types account for more than 1%, and the rest are mostly characteristic fatty acids present in trace amounts [32]. There are significant differences in the effects of various types of fatty acids on human health. PUFAs such as alpha-linolenic acid, gamma-linolenic acid, and eicosapentaenoic acid are essential functional fatty acids that offer multiple physiological benefits, including lipid regulation, antioxidant activity, and cardiovascular health maintenance, making them important bioactive nutritional components in milk. In contrast, excessive intake of SFAs like palmitic acid and myristic acid may increase metabolic risks such as lipid abnormalities, which are detrimental to overall health [33]. In this study, the addition of ADY II significantly reduced the fatty acid content in milk. A previous study showed that adding 0.5 g of live yeast to the diet of Ayrshire dairy cows had no effect on the milk production of dairy cows and had no effect on the proportion of major fatty acids in milk [34]. Another study showed that adding yeast to the diet of dairy goats significantly reduced the concentrations of medium- and short-chain fatty acids and unsaturated fatty acids in milk [35]. Moreover, studies have shown that rumen biohydrogenation intermediates can regulate mammary lipid synthesis [36]. In this study, the addition of ADY II significantly reduced the fatty acid content in milk. This may be due to the ADY altering rumen fermentation patterns and microbial community structure, regulating the biohydrogenation process of fatty acids, and changing their absorption and metabolism. It could also affect lipid metabolism in dairy cows and the mammary gland’s capacity for fatty acid synthesis and deposition, thereby modifying milk fatty acid concentrations. The specific mechanisms require further investigation.

Nutrient digestibility refers to the percentage of the number of nutrients in feed that are digested and absorbed by animals to the total number of such substances ingested [37]. It is a core indicator for evaluating the nutritional value of feed and the digestive capacity of animals [38,39]. In this study, the addition of ADY II significantly improved the apparent digestibility of CP in dairy cows, consistent with findings by Salah et al. (2026) [40], who reported that supplementing yeast in dairy cow diets enhances protein apparent digestibility by reducing fecal nitrogen loss. The underlying microscopic mechanism is supported by Ma et al. (2021) [41], who demonstrated that ADY II significantly reduces ruminal ammonia-N concentration while simultaneously increasing MCP production, indicating more efficient conversion of free nitrogen into MCP. Furthermore, Dai et al. (2023) [42] confirmed using an in vitro method that yeast supplementation increased CP digestibility by 9.6%, and sequencing analysis revealed enhanced proliferation of beneficial facultative bacteria such as *Selenomonas*. In this study, HADYII increased the apparent CP digestibility, but NH_3_-N and MCP concentrations did not change significantly; the specific mechanism remains undetermined.

Rumen fermentation parameters are key indicators for evaluating the homeostasis of the rumen environment and the efficiency of nutrient digestion and metabolism in ruminant animals [43]. Ruminal pH reflects the balance between acid accumulation and buffering and is an important determinant of the environment in which rumen microorganisms function [44]. In the present study, ruminal pH did not differ significantly among treatments when measured 2 h after morning feeding. Garnsworthy et al. (2025) [3] reported no significant effect on ruminal pH when 50 Holstein cows received either a control diet or 10 g/day of live yeast. This is consistent with the results of the present study. Ruminal NH_3_-N provides a nitrogen source for microbial growth, and its concentration reflects the balance between nitrogen degradation, microbial uptake, absorption, and passage [45]. In this study, NH_3_-N concentration did not differ significantly among the four groups. This result agrees with Li et al. (2021) [46], who evaluated ADY supplementation at 0, 10, 20, and 30 g/day in 60 early-lactating Holstein cows and found no significant effect on ruminal ammonia concentration. MCP represents nitrogen incorporated into microbial biomass and contributes substantially to the amino acids available to the host after intestinal digestion [47]. Ruminal MCP concentration did not differ significantly among treatments in the present study. This finding aligns with Salah et al. (2026) [40], who reported no significant effect of supplementing dairy cow diets with 5 g of ADY on ruminal MCP synthesis. VFAs are major products of ruminal carbohydrate fermentation and provide energy for the host, while acetate also serves as a precursor for milk fat synthesis [48]. Li et al. (2023) [5] reported that dietary supplementation with 10 or 20 g/day of live *Saccharomyces cerevisiae* significantly increased ruminal VFA concentrations in heat-stressed Holstein cows. This finding is consistent with the present study, in which both LADY II and HADY II increased ruminal TVFA and acetate concentrations. These increases may partly reflect the greater availability of fermentable substrates associated with the higher feed intake observed in cows receiving ADY II. In addition, ADY II may have modulated ruminal microbial activity, potentially promoting VFA formation. This explanation is supported by in vitro evidence showing that certain live yeast strains accelerate oxygen consumption, creating more favorable conditions for anaerobic microbial activity [49]. However, the fermentation parameters measured in the present study cannot identify the specific microbial groups or metabolic pathways involved, and further research is needed to clarify these mechanisms.

Routine hematological tests provide information on the physiological status of dairy cows and help identify abnormalities associated with nutrition, inflammation, and blood system disorders. Red blood cell count, hemoglobin concentration, and packed cell volume describe the erythrocytic component and reflect the oxygen-carrying capacity of the blood, while platelet count is related to hemostatic function. White blood cell count and its differential composition reflect the status of circulating immune cells, with granulocytes involved in innate defense and inflammatory responses [50]. Studies have shown that supplementing lactating Holstein cows with 10–30 g of hydrolyzed yeast had no significant effect on RBC, WBC, HGB, or HCT [51]. Additionally, supplementing growing heifers’ diets with live yeast reduced circulating neutrophil count from 4.27 to 3.81 × 10^9^/L, whereas RBC, HGB, HCT, or PLT showed no significant differences among treatments [52]. These findings are largely consistent with the present study. In this study, there were no significant differences in red blood cell count, hemoglobin concentration, packed cell volume, or platelet count among treatment groups. Leukocyte and granulocyte counts in LADY II and HADY II were lower than those in ADY I, but neither differed significantly from the CON. However, blood samples were collected only at the end of the experiment, lacking baseline data, thus preventing assessment of individual cow changes over time. Furthermore, immune cell function and inflammatory biomarkers were not evaluated. Therefore, future research should combine repeated hematological testing with functional immune analysis to clarify the biological significance and underlying mechanisms of these differences.

From a practical perspective, supplementation with ADY II at 10 g/cow/day increased milk yield by 2.47 kg/day and DMI by 1.86 kg/day compared with the control. These findings may inform supplementation practices on dairy farms with similar feeding and management conditions. Although ADY II supplementation at 5 g/cow/day also increased DMI and ruminal TVFA and acetate concentrations, it did not significantly increase milk yield relative to the control. Therefore, ADY II at 10 g/cow/day warrants further evaluation under commercial farm conditions, although a more comprehensive dose–response trial is needed to determine the optimal inclusion level. This study also has certain limitations. ADY I was evaluated only at 5 g/cow/day, whereas ADY II was evaluated at both 5 and 10 g/cow/day. Thus, the effects of the two products could be compared at 5 g/cow/day, but the absence of a 10 g/cow/day ADY I treatment prevented a direct comparison at the higher inclusion level. In addition, hematological parameters were measured only at the end of the study, without baseline measurements. This limited the assessment of pre-existing differences among treatment groups and prevented evaluation of changes from baseline. Future studies should collect blood samples before supplementation and during the trial to allow baseline adjustment and assessment of changes over time. Future studies should evaluate both products at matched doses across a broader dose range and integrate multi-omics analyses with functional validation to clarify the mechanisms through which ADY II may influence feed intake, lactation performance, and ruminal fermentation in dairy cows.

## 5. Conclusions

Under the experimental conditions, ADY II (Actisaf Sc48) at 10 g per cow per day increased milk yield and apparent CP digestibility compared to the CON and ADY I (RF7) at 5 g per cow per day. Both doses of ADY II significantly increased total DMI, ruminal TVFA, and acetate concentration, and altered the fatty acid composition in milk. However, since a treatment group with ADY I at 10 g per cow per day was not included, a direct comparison between the two products at higher doses could not be made, limiting the ability to clearly attribute changes in dairy performance to strain-specific effects. Additionally, the lack of baseline data from single blood tests restricted the generalizability of results and interpretation regarding animal health. Future studies should compare different products using identical dosages, incorporate baseline data, and further investigate the underlying mechanisms and economic benefits to determine the optimal supplementation level.

## Figures and Tables

**Table 1:** Dietary composition and nutrient levels of dairy cows (DM basis).

Feed Ingredients	Proportion (%)	Nutrient Composition	Content
Corn silage	51.34	DM (%)	52.71
Steam-flaked corn	8.13	CP (%)	16.61
Corn	5.35	NDF (%)	34.13
Alfalfa	7.06	ADF (%)	18.18
Brewer’s grains	10.70	EE (%)	4.98
Soybean meal	8.56	Ca (%)	0.77
Cottonseed	2.78	P (%)	0.32
Soybean hulls	2.14	ASH (%)	6.61
Molasses	2.14	NEL ^1^, MJ/kg DM	6.53
Baking soda	0.32		
Premix	1.50		

One kg premix contained the following: VA 200,000 IU, VD 60,000 IU, Fe 400 mg, Cu 450 mg, Zn 2000 mg, Mn 550 mg, Se 15 mg, Co 20 mg. DM = dry matter; CP = crude protein; EE = ether extract; NDF = neutral detergent fiber; ADF = acid detergent fiber; ASH = ash (crude ash); NEL = net energy for lactation. ^1^ The NEL of this dairy cow diet was estimated based on data from NRC (2001) and standard feed composition tables [9].

**Table 2:** Effects of different ADY on milk yield and milk composition in dairy cows.

Item	Treatment				SEM	*p*-Value		
	CON	ADY I	LADY II	HADY II		Treatment	Time	Treatment × Time
Milk yield, kg/d	42.87 ^a^	43.28 ^a^	44.42 ^ab^	45.34 ^bc^	0.423	0.021	<0.001	0.957
DMI, kg/d	26.72 ^a^	27.65 ^b^	28.15 ^bc^	28.58 ^c^	0.160	<0.001	<0.001	<0.001
Milk composition								
Milk fat, %	4.49	4.35	4.29	4.22	0.049	0.275	0.942	0.502
Milk protein, %	3.19	3.32	3.23	3.25	0.036	0.101	<0.001	0.910
Lactose, %	5.04 ^ab^	4.95 ^a^	5.05 ^ab^	5.12 ^b^	0.023	0.042	0.002	0.224
Total solids, %	13.23	13.30	13.06	13.06	0.062	0.433	0.324	0.838
MUN, mg N/dL	18.41	19.24	19.79	18.73	0.448	0.128	<0.001	0.197
SCC, 10^3^ cells/mL	286.78	201.06	315.00	140.28	74.729	0.833	0.074	0.488

Within a row, means with no common superscript letters differ significantly (*p* < 0.05). DMI = dry matter intake; MUN = milk urea nitrogen; SCC = somatic cell count; CON = control; ADY I = active dry yeast I; LADY II = low-dose active dry yeast II; HADY II = high-dose active dry yeast II; SEM = standard error of the mean. CON = fed the basal diet. ADY I = fed the basal diet supplemented with 5 g/d of RF7. LADY II = fed the basal diet supplemented with 5 g/d of Actisaf Sc48. HADY II = fed the basal diet supplemented with 10 g/d of Actisaf Sc48.

**Table 3:** Effects of different ADY on milk fatty acids.

Fatty Acid (mg/mL)	Treatment				SEM	*p*-Value		
	CON	ADY I	LADY II	HADY II		Treatment	Time	Treatment × Time
Butyric acid	0.018 ^a^	0.044 ^b^	0.013 ^a^	0.004 ^a^	0.004	<0.001	0.032	<0.001
Caproic acid	0.166 ^a^	0.193 ^a^	0.072 ^b^	0.051 ^b^	0.017	<0.001	0.012	<0.001
Caprylic acid	0.225 ^a^	0.249 ^a^	0.115 ^b^	0.114 ^b^	0.016	<0.001	0.003	<0.001
Capric acid	0.559 ^a^	0.633 ^a^	0.371 ^b^	0.379 ^b^	0.035	0.001	0.010	<0.001
Undecanoic acid	0.021 ^a^	0.032 ^b^	0.012 ^a^	0.015 ^a^	0.002	<0.001	0.018	<0.001
Lauric acid	0.641 ^ab^	0.739 ^a^	0.455 ^b^	0.464 ^b^	0.039	0.003	0.013	<0.001
Tridecanoic acid	0.030 ^ab^	0.039 ^a^	0.017 ^c^	0.020 ^bc^	0.002	<0.001	0.010	<0.001
Myristic acid	2.015 ^a^	2.037 ^a^	1.317 ^b^	1.385 ^b^	0.115	0.002	0.007	<0.001
Myristoleic acid	0.176 ^a^	0.190 ^a^	0.113 ^b^	0.105 ^b^	0.010	<0.001	0.001	<0.001
Pentadecenoic acid	0.214 ^a^	0.252 ^a^	0.120 ^b^	0.138 ^b^	0.015	<0.001	0.006	<0.001
cis-10-Pentadecenoic acid	0.027	0.028	0.025	0.021	0.002	0.168	0.013	0.001
Palmitic acid	6.730 ^a^	7.042 ^a^	4.285 ^b^	4.507 ^b^	0.409	0.001	0.002	<0.001
Palmitoleic acid	0.288 ^a^	0.353 ^a^	0.180 ^b^	0.189 ^b^	0.019	<0.001	0.002	<0.001
Heptadecanoic acid	0.100 ^a^	0.101 ^a^	0.060 ^b^	0.065 ^b^	0.006	<0.001	0.003	<0.001
cis-10-Heptadecenoic acid	0.032 ^a^	0.038 ^a^	0.017 ^b^	0.021 ^b^	0.002	<0.001	0.040	<0.001
Stearic acid	1.916 ^a^	1.726 ^ab^	1.309 ^b^	1.283 ^b^	0.108	0.004	0.004	<0.001
Elaidic acid	0.121	0.129	0.110	0.087	0.012	0.674	0.153	0.420
Oleic acid	3.401 ^a^	3.181 ^a^	2.123 ^b^	2.095 ^b^	0.198	<0.001	0.001	<0.001
Linolelaidic acid	0.135 ^a^	0.116 ^ab^	0.085 ^c^	0.088 ^bc^	0.007	<0.001	0.001	<0.001
Linoleic acid	0.636 ^a^	0.616 ^a^	0.433 ^b^	0.448 ^b^	0.033	0.001	<0.001	<0.001
Arachidic acid	0.010 ^a^	0.009 ^a^	0.007 ^b^	0.007 ^b^	<0.001	<0.001	0.012	<0.001
γ-Linolenic acid	0.079 ^a^	0.074 ^a^	0.053 ^b^	0.057 ^b^	0.004	0.001	0.001	<0.001
cis-11-Eicosenoic acid	0.026 ^a^	0.024 ^ab^	0.017 ^b^	0.017 ^b^	0.002	0.007	0.007	<0.001
α-Linolenic acid	0.017 ^a^	0.014 ^a^	0.009 ^b^	0.009 ^b^	0.001	<0.001	0.006	<0.001
Heneicosanoic acid	0.007 ^a^	0.006 ^ab^	0.005 ^b^	0.005 ^b^	<0.001	0.009	0.016	0.004
cis-11,14-Eicosadienoic acid	0.004	0.003	0.003	0.003	<0.001	0.086	0.170	<0.001
Behenic acid	0.034 ^a^	0.030 ^ab^	0.023 ^b^	0.024 ^b^	0.002	0.001	0.005	<0.001
cis-8,11,14-Eicosatrienoic acid	0.037 ^a^	0.039 ^a^	0.024 ^b^	0.027 ^b^	0.002	0.001	0.002	<0.001
Erucic acid	0.004	0.002	0.004	0.003	<0.001	0.097	0.008	<0.001
cis-11,14,17-Eicosatrienoic acid	0.008 ^a^	0.007 ^a^	0.007 ^ab^	0.005 ^b^	<0.001	0.001	0.089	<0.001
Arachidonic acid	0.013	0.010	0.010	0.009	0.001	0.051	0.200	<0.001
Tricosanoic acid	0.005	0.003	0.004	0.004	<0.001	0.127	0.242	0.048
cis-13,16-Docosadienoic acid	0.003	0.002	0.002	0.002	<0.001	0.443	0.414	0.019
Lignoceric acid	0.009 ^a^	0.009 ^a^	0.007^b^	0.007 ^ab^	<0.001	0.011	0.916	0.002
cis-5,8,11,14,17-Eicosapentaenoic acid	0.015 ^a^	0.014 ^a^	0.010^b^	0.011 ^b^	0.001	0.001	0.011	<0.001
Nervonic acid	0.001	0.001	0.001	0.001	<0.001	0.084	0.504	0.076
cis-4,7,10,13,16,19-Docosahexaenoic acid	0.008	0.009	0.005	0.005	0.001	0.054	0.128	<0.001

Within a row, means with no common superscript letters differ significantly (*p* < 0.05). CON = control; ADY I = active dry yeast I; LADY II = low-dose active dry yeast II; HADY II = high-dose active dry yeast II; SEM = standard error of the mean. CON = fed the basal diet. ADY I = fed the basal diet supplemented with 5 g/d of RF7. LADY II = fed the basal diet supplemented with 5 g/d of Actisaf Sc48. HADY II = fed the basal diet supplemented with 10 g/d of Actisaf Sc48.

**Table 4:** Effects of ADY on apparent total-tract digestibility of dairy cows.

Item	Treatment				SEM	*p*-Value
	CON	ADY I	LADY II	HADY II		
CP, %	70.73 ^a^	72.13 ^a^	73.75 ^ab^	74.42 ^bc^	0.889	0.049
EE, %	72.88	72.85	73.16	74.12	0.947	0.392
NDF, %	55.03	55.14	55.16	56.23	0.832	0.471
ADF, %	41.21	42.56	44.99	45.06	2.213	0.822

Within a row, means with no common superscript letters differ significantly (*p* < 0.05). CP = crude protein; EE = ether extract; NDF = neutral detergent fiber; ADF = acid detergent fiber; CON = control; ADY I = active dry yeast I; LADY II = low-dose active dry yeast II; HADY II = high-dose active dry yeast II; SEM = standard error of the mean. CON = fed the basal diet. ADY I = fed the basal diet supplemented with 5 g/d of RF7. LADY II = fed the basal diet supplemented with 5 g/d of Actisaf Sc48. HADY II = fed the basal diet supplemented with 10 g/d of Actisaf Sc48.

**Table 5:** Effects of ADY on rumen fermentation of dairy cows.

Item	Treatment				SEM	*p*-Value
	CON	ADY I	LADY II	HADY II		
pH	6.56	6.37	6.11	6.19	0.105	0.182
NH_3_-N, mg/dL	22.10	21.24	19.38	18.77	1.545	0.690
MCP, mg/dL	61.16	69.86	79.11	88.64	7.361	0.320
TVFA, mmol/L	113.19 ^b^	122.96 ^b^	160.85 ^a^	163.81 ^a^	9.253	0.025
Acetate, mmol/L	92.46 ^b^	101.55 ^b^	134.77 ^a^	135.65 ^a^	7.924	0.019
Propionate, mmol/L	15.74	16.32	20.19	22.00	1.601	0.185
Butyrate, mmol/L	4.21	4.38	5.06	5.47	0.742	0.797
Isobutyrate, mmol/L	0.69	0.67	0.63	0.66	0.068	0.937
A/P	5.87	6.22	6.67	6.17	0.413	0.514

Within a row, means with no common superscript letters differ significantly (*p* < 0.05). NH_3_-N = ammonia nitrogen; MCP = microbial protein; TVFA = total volatile fatty acids; A/P = acetate-to-propionate ratio. CON = control; ADY I = active dry yeast I; LADY II = low-dose active dry yeast II; HADY II = high-dose active dry yeast II; SEM = standard error of the mean. CON = fed the basal diet. ADY I = fed the basal diet supplemented with 5 g/d of RF7. LADY II = fed the basal diet supplemented with 5 g/d of Actisaf Sc48. HADY II = fed the basal diet supplemented with 10 g/d of Actisaf Sc48.

**Table 6:** Effects of ADY on hematological profile of dairy cows.

Item	Treatment				SEM	*p*-Value
	CON	ADY I	LADY II	HADY II		
WBC,10^9^/L	77.69 ^ab^	86.62 ^a^	62.10 ^b^	55.10 ^b^	4.094	0.020
Lymph, 10^9^/L	45.76	50.67	37.87	33.08	2.822	0.111
Mon, 10^9^/L	5.94	4.99	3.15	3.78	0.399	0.060
Gran, 10^9^/L	25.99 ^ab^	30.96 ^a^	20.81 ^b^	18.74 ^b^	1.733	0.046
Lymph%	59.42	57.35	61.26	59.18	1.635	0.875
Mon%	7.44	5.83	5.37	7.36	0.454	0.260
Gran%	33.06	36.82	33.37	33.51	1.490	0.799
RBC, 10^12^/L	6.17	6.18	6.14	6.36	0.087	0.805
HGB, g/L	116.56	117.30	113.80	116.80	1.397	0.821
HCT, %	32.32	32.21	31.62	32.77	0.376	0.764
MCV, fL	52.56	52.26	51.65	50.73	0.574	0.705
MCH, pg	18.86	18.94	18.51	18.22	0.148	0.290
MCHC, g/L	359.89	363.80	359.60	373.80	5.201	0.760
RDW, %	15.91	15.56	15.86	15.66	0.133	0.781
PLT, 10^9^/L	584.56	517.30	584.40	527.00	23.918	0.650
MPV, fL	5.80	5.96	5.99	6.11	0.075	0.564
PDW	16.48	16.85	16.31	16.57	0.078	0.090
PCT, %	0.34	0.28	0.32	0.32	0.017	0.716

Within a row, means with no common superscript letters differ significantly (*p* < 0.05). WBC = white blood cell; Lymph = lymphocyte; Mon = monocyte; Gran = granulocyte; Lymph% = lymphocyte percentage; Mon% = monocyte percentage; Gran% = granulocyte percentage; RBC = red blood cell; HGB = hemoglobin; HCT = hematocrit; MCV = mean corpuscular volume; MCH = mean corpuscular hemoglobin; MCHC = mean corpuscular hemoglobin concentration; RDW = red blood cell distribution width; PLT = platelet; MPV = mean platelet volume; PDW = platelet distribution width; PCT = plateletcrit; CON = control; ADY I = active dry yeast I; LADY II = low-dose active dry yeast II; HADY II = high-dose active dry yeast II; SEM = standard error of the mean. CON = fed the basal diet. ADY I = fed the basal diet supplemented with 5 g/d of RF7. LADY II = fed the basal diet supplemented with 5 g/d of Actisaf Sc48. HADY II = fed the basal diet supplemented with 10 g/d of Actisaf Sc48.

## Data Availability

The original contributions presented in this study are included in the article. Further inquiries can be directed to the corresponding authors.

## References

[1] Chaucheyras-Durand F., Walker N.D., Bach A. (2008). Effects of active dry yeasts on the rumen microbial ecosystem: Past, present and future. Anim. Feed Sci. Technol..

[2] Amin A.B., Mao S. (2021). Influence of yeast on rumen fermentation, growth performance and quality of products in ruminants: A review. Anim. Nutr..

[3] Garnsworthy P.C., Saunders N., Goodman J.R., Algherair I.H., Ambrose J.D. (2025). Effects of live yeast on milk yield, feed efficiency, methane emissions and fertility of high-yielding dairy cows. Animal.

[4] Kumprechtová D., Legendre H., Kadek R., Nenov V., Briche M., Salah N., Illek J. (2024). Dose effect of Actisaf Sc 47 yeast probiotic (*Saccharomyces cerevisiae*) supplementation on production, reproduction, and negative energy balance in early lactation dairy cows. Transl. Anim. Sci..

[5] Li Z., Fan Y., Bai H., Zhang J., Mao S., Jin W. (2023). Live yeast supplementation altered the bacterial community’s composition and function in rumen and hindgut and alleviated the detrimental effects of heat stress on dairy cows. J. Anim. Sci..

[6] Marden J.P., Julien C., Monteils V., Auclair E., Moncoulon R., Bayourthe C. (2008). How does live yeast differ from sodium bicarbonate to stabilize ruminal pH in high-yielding dairy cows?. J. Dairy Sci..

[7] Takiya C.S., Chesini R.G., de Freitas A.C., Grigoletto N.T.S., Vieira D.J.C., Poletti G., Martins N.P., Sbaralho O.P., Roth N., Acedo T. (2024). Dietary supplementation with live or autolyzed yeast: Effects on performance, nutrient digestibility, and ruminal fermentation in dairy cows. J. Dairy Sci..

[8] Jiang Y., Ogunade I.M., Arriola K.G., Qi M., Vyas D., Staples C.R., Adesogan A.T. (2017). Effects of the dose and viability of *Saccharomyces cerevisiae*. 2. Ruminal fermentation, performance of lactating dairy cows, and correlations between ruminal bacteria abundance and performance measures. J. Dairy Sci..

[9] National Research Council (2001). Nutrient Requirements of Dairy Cattle.

[10] Bondan C., Folchini J.A., Noro M., Machado K.M., Muhls E., González F.H.D. (2019). Variation of cow’s milk composition across different daily milking sessions and feasibility of using a composite sampling. Cienc. Rural..

[11] Van Keulen J., Young B.A. (1977). Evaluation of acid-insoluble ash as a natural marker in ruminant digestibility studies. J. Anim. Sci..

[12] (2016). National Food Safety Standard—Determination of Fatty Acids in Foods.

[13] (1986). Method for the Determination of Moisture in Feedstuffs.

[14] (1994). Method for the Determination of Crude Protein in Feedstuffs.

[15] (1994). Method for the Determination of Crude Fat in Feedstuffs.

[16] (1992). Method for the Determination of Crude Ash in Feedstuffs.

[17] Van Soest P.J., Robertson J.B., Lewis B.A. (1991). Methods for dietary fiber, neutral detergent fiber, and nonstarch polysaccharides in relation to animal nutrition. J. Dairy Sci..

[18] Bradford M.M. (1976). A rapid and sensitive method for the quantitation of microgram quantities of protein utilizing the principle of protein-dye binding. Anal. Biochem..

[19] Broderick G.A., Kang J.H. (1980). Automated simultaneous determination of ammonia and total amino acids in ruminal fluid and in vitro media. J. Dairy Sci..

[20] Herman N., Trumel C., Geffré A., Braun J.P., Thibault M., Schelcher F., Bourgès-Abella N. (2018). Hematology reference intervals for adult cows in France using the Sysmex XT-2000iV analyzer. J. Vet. Diagn. Investig..

[21] Chen H., Qiu D., Miao X., Yang W., He Q., Ren H., Zhang L., Ruan H., Zhang J., Zhang N. (2025). Zearalenone Depresses Lactation Capacity Through the ROS-Mediated PI3K/AKT Pathway. Animals.

[22] Wang S., Long T., Wei X., Guo Q., Guo H., Shen W., Gu Z. (2026). Research on the Assessment of Dairy Cow Dry Matter Intake Using ITSO-Optimized Stacking Ensemble Learning. Animals.

[23] Wang D., Liang G., Wang B., Sun H., Liu J., Guan L.L. (2016). Systematic microRNAome profiling reveals the roles of microRNAs in milk protein metabolism and quality: Insights on low-quality forage utilization. Sci. Rep..

[24] Suayroop N., Khanthusaeng V., Kraisoon A., Bunma T., Nabthonglang J., Navanukraw P., Haitook T., Cherdthong A., Navanukraw C. (2026). Long-Term *Saccharomyces cerevisiae* Supplementation Enhances Milk Yield and Reproductive Performance in Lactating Dairy Cows on Smallholder Farms. Animals.

[25] Cabrita A.R.J., Carvalheira J., Fonseca A.J.M. (2025). Live Yeast Supplementation Attenuates the Effects of Heat Stress in Dairy Cows. Vet. Sci..

[26] Soder K.J., Holden L.A. (1999). Dry matter intake and milk yield and composition of cows fed yeast prepartum and postpartum. J. Dairy Sci..

[27] Malik M.I., Raboisson D., Zhang X., Sun X. (2023). Effects of dietary chromium supplementation on dry matter intake and milk production and composition in lactating dairy cows: A meta-analysis. Front. Vet. Sci..

[28] Ogbuewu I.P., Mbajiorgu C.A. (2022). Meta-analytic effect of *Saccharomyces cerevisiae* on dry matter intake, milk yield and components of lactating goats. Front. Vet. Sci..

[29] Poppy G.D., Rabiee A.R., Lean I.J., Sanchez W.K., Dorton K.L., Morley P.S. (2012). A meta-analysis of the effects of feeding yeast culture produced by anaerobic fermentation of *Saccharomyces cerevisiae* on milk production of lactating dairy cows. J. Dairy Sci..

[30] Várhidi Z., Máté M., Ózsvári L. (2022). The use of probiotics in nutrition and herd health management in large Hungarian dairy cattle farms. Front. Vet. Sci..

[31] Torres-Gonzalez M., Rice Bradley B.H. (2023). Whole-Milk Dairy Foods: Biological Mechanisms Underlying Beneficial Effects on Risk Markers for Cardiometabolic Health. Adv. Nutr..

[32] Zhao X., Song Y., Zhang Y., Cai G., Xue G., Liu Y., Chen K., Zhang F., Wang K., Zhang M. (2023). Predictions of Milk Fatty Acid Contents by Mid-Infrared Spectroscopy in Chinese Holstein Cows. Molecules.

[33] Yao D., Sun L.-C., Zhang L.-J., Chen Y.-L., Miao S., Cao M.-J., Lin D. (2024). Emulsion Structural Remodeling in Milk and Its Gelling Products: A Review. Gels.

[34] Bayat A.R., Kairenius P., Stefański T., Leskinen H., Comtet-Marre S., Forano E., Chaucheyras-Durand F., Shingfield K.J. (2015). Effect of camelina oil or live yeasts (*Saccharomyces cerevisiae*) on ruminal methane production, rumen fermentation, and milk fatty acid composition in lactating cows fed grass silage diets. J. Dairy Sci..

[35] Sulistyowati E., Sudarman A., Wiryawan K.G., Toharmat T. (2013). Quality of milk fatty acid during late lactation in dairy goat fed on PUFA-diet supplemented with yeast and *Curcuma xanthorrhiza* Roxb. J. Indones. Trop. Anim. Agric..

[36] Ibeagha-Awemu E.M., Li R., Ammah A.A., Dudemaine P.-L., Bissonnette N., Benchaar C., Zhao X. (2016). Transcriptome adaptation of the bovine mammary gland to diets rich in unsaturated fatty acids shows greater impact of linseed oil over safflower oil on gene expression and metabolic pathways. BMC Genom..

[37] Watts A., Mcclintock B., Lawrence M.C. (2007). Developments in Aquaculture and Fisheries Science.

[38] Zhang F., Jin E., Liu X., Ji X., Hu H. (2022). Effect of Dietary Fructus mume and *Scutellaria baicalensis* Georgi on the Fecal Microbiota and Its Correlation with Apparent Nutrient Digestibility in Weaned Piglets. Animals.

[39] Guo B., He X., Ge C., Xue M., Wang J., Longshaw M., Wang J., Liang X. (2022). A Natural Gas Fermentation Bacterial Meal (FeedKind^®^) as a Functional Alternative Ingredient for Fishmeal in Diet of Largemouth Bass, *Micropterus salmoides*. Antioxidants.

[40] Salah N., Gestes B., Ly P., Blancou A., Hadjeba K., Schulthess J., Duclos J., Pinloche E. (2026). Reducing Protein Content with and Without Yeast Probiotic Actisaf Sc 47 Supplementation in the Diet of Dairy Cow: Effects on Nitrogen Use, Digestibility, and Rumen Microbial Protein. Animals.

[41] Ma J., Wang C., Wang Z., Cao G., Hu R., Wang X., Zou H., Kang K., Peng Q., Xue B. (2021). Active dry yeast supplementation improves the growth performance, rumen fermentation, and immune response of weaned beef calves. Anim. Nutr..

[42] Dai D., Liu Y., Kong F., Guo C., Dong C., Xu X., Li S., Wang W. (2023). *Saccharomyces cerevisiae* Culture’s Dose–Response Effects on Ruminal Nutrient Digestibility and Microbial Community: An In Vitro Study. Fermentation.

[43] Yi X., Wu B., Ma J., Cui X., Deng Z., Hu S., Li W., A R., Li X., Meng Q. (2023). Effects of Dietary Capsaicin and *Yucca schidigera* Extracts as Feed Additives on Rumen Fermentation and Microflora of Beef Cattle Fed with a Moderate-Energy Diet. Fermentation.

[44] Ran T., Xu Z., Yang W., Liu D., Wu D. (2024). Partially substituting alfalfa hay with hemp forage in the diet of goats improved feed efficiency, ruminal fermentation pattern and microbial profiles. Anim. Nutr..

[45] Reynolds C.K., Kristensen N.B. (2008). Nitrogen recycling through the gut and the nitrogen economy of ruminants: An asynchronous symbiosis. J. Anim. Sci..

[46] Li Y., Shen Y., Niu J., Guo Y., Pauline M., Zhao X., Li Q., Cao Y., Bi C., Zhang X. (2021). Effect of active dry yeast on lactation performance, methane production, and ruminal fermentation patterns in early-lactating Holstein cows. J. Dairy Sci..

[47] Pan Y., Sun G., Li G., Chen S., Liu H., Li H., Mei C., Yang W., Zan L. (2025). Sex-specific microbiota associations with backfat thickness, eye muscle area, and rumen fermentation in Qinchuan cattle. BMC Microbiol..

[48] Schären M., Seyfang G.M., Steingass H., Dieho K., Dijkstra J., Hüther L., Frahm J., Beineke A., von Soosten D., Meyer U. (2016). The effects of a ration change from a total mixed ration to pasture on rumen fermentation, volatile fatty acid absorption characteristics, and morphology of dairy cows. J. Dairy Sci..

[49] Newbold C.J., Wallace R.J., McIntosh F.M. (1996). Mode of action of the yeast *Saccharomyces cerevisiae* as a feed additive for ruminants. Br. J. Nutr..

[50] Roland L., Drillich M., Iwersen M. (2014). Hematology as a diagnostic tool in bovine medicine. J. Vet. Diagn. Investig..

[51] Nikkhah M., Chamani M., Sadeghi A.A., Hasan-Sajedi R. (2023). Effects of enzymatically hydrolyzed yeast supplementation on blood attributes, antioxidant status and gene expression of cytokines in vaccinated dairy cows. Anim. Biotechnol..

[52] Broadway P.R., Carroll J.A., Burdick Sanchez N.C., Cravey M.D., Corley J.R. (2020). Some Negative Effects of Heat Stress in Feedlot Heifers May Be Mitigated via Yeast Probiotic Supplementation. Front. Vet. Sci..

